# Correction: Bioenergetic function is decreased in peripheral blood mononuclear cells of veterans with Gulf War Illness

**DOI:** 10.1371/journal.pone.0302501

**Published:** 2024-04-18

**Authors:** Joel N. Meyer, William K. Pan, Ian T. Ryde, Thomas Alexander, Jacquelyn C. Klein-Adams, Duncan S. Ndirangu, Michael J. Falvo

Fig 3A shows specific OCR-related parameters for each of the three visits (corresponding to the three visits shown in panels B-F). However, this graph might be confusing for readers accustomed to typical Seahorse mitochondrial stress test graphs, which illustrate different OCR-related parameters (specifically, OCR at baseline and after each drug injection), as well as average values for all measurements at each timepoint in the Seahorse runs. Additionally, the original Fig 3A depicts "Basal-ATP" for the second series of data points, rather than the more common "ATP-linked" (post-oligomycin injection) values.

A more typical Seahorse mitochondrial stress test graph (Fig 3) with Seahorse analyses to aid understanding is provided here. This does not alter any major conclusions but necessitates disregarding the following sentence in the text: "However, we note that differences in OCR after FCCP injection were much larger at the second and third readings after FCCP injection, and that OCR declined consistently after FCCP injection for the GWI population, but not the non-GWI population."

Please view the correct Fig 3 here.

**Fig 3:**
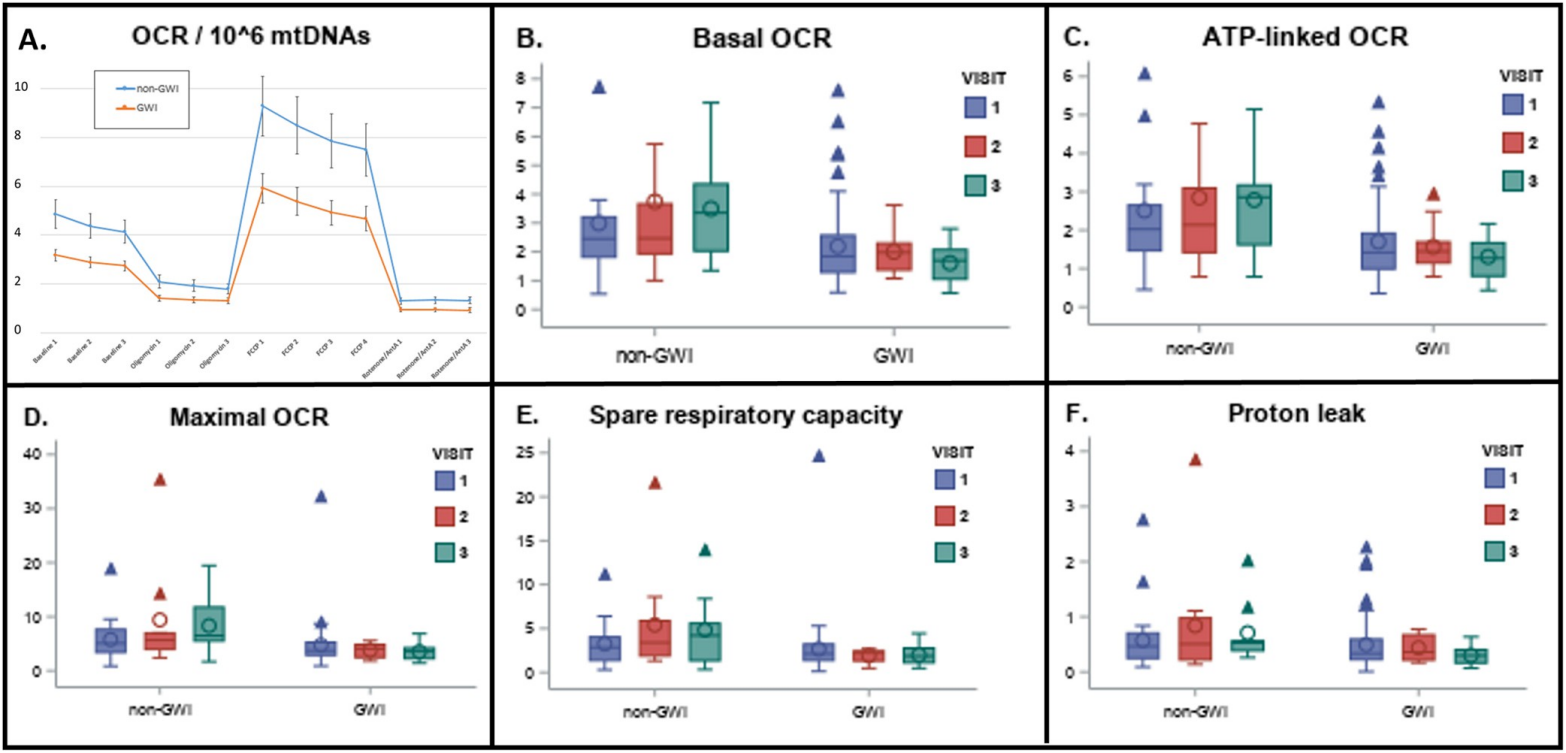
Decreased mitochondrial respiratory function in GWI. A) Average (with standard errors of the mean) oxygen consumption in all samples measured from veterans with and without GWI. Values are normalized to 1,000,000 mtDNA copies. The “basal” reading reflects the amount of oxygen being consumed by cells without the addition of any drugs. ATP-linked OCR is the amount of oxygen consumed upon injection of oligomycin, which inhibits ATP synthesis and therefore blocks oxygen consumption associated with converting ADP to ATP. Maximal respiration is respiration after injection of FCCP, which uncouples mitochondria. Non-mitochondrial respiration is the amount of respiration remaining after the electron transport chain is entirely inhibited with a combination of rotenone and antimycin A. See Fig 1 for a schematic illustration of the effect of the drugs. Spare respiratory capacity is the differences between maximal and basal; proton leak is the difference between ATP-linked and non-mitochondrial. In Panel A, the graph includes all Seahorse runs, i.e., it includes one analysis for each participant who made one visit, and two or three for each volunteer who visited 2 or three times. Specific OCR-related parameters of particular interest are graphed by Kansas status and visit in panels B-F.
