# Supplementary material for: Bioenergetic function is decreased in peripheral blood mononuclear cells of veterans with Gulf War Illness
Source: PLoS One. 2023 Nov 1;18(11):e0287412. doi: 10.1371/journal.pone.0287412 (PMC10619881; doi:10.1371/journal.pone.0287412)
Supplement: S1 Fig — Shown are mean effects and 95% confidence intervals for days of chemical exposures, adjusting for GWI status. (PDF) [file pone.0287412.s001.pdf]

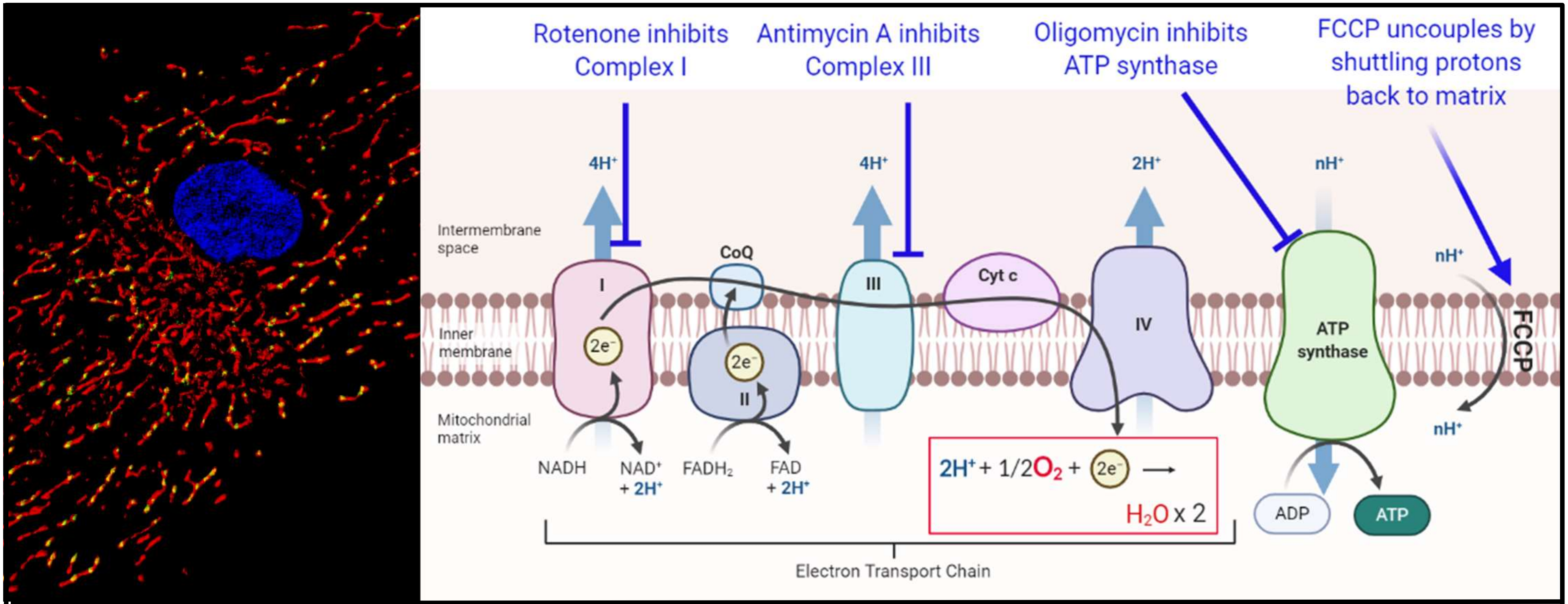

Figure 1. Schematic illustrating mitochondrial parameters analyzed: mtDNA copy number (CN), mtDNA damage, mitochondrial and non-mitochondrial oxygen consumption. Left panel from Meyer et al., 2013 shows the many mtDNAs in a cell in yellow (mitochondria in red, nucleus in blue). The number of mtDNAs is stress-responsive. Decreased number or damage to mtDNA could interfere with proper assembly of the electron transport chain (right panel), because components of Complexes I, III, IV, and ATP synthase are encoded in mtDNA; increased mtDNA CN may indicate a stress-responsive attempt to compensate for decreased function. The electron transport chain converts food-derived fuel to a proton gradient by pumping protons out of the matrix. ATP synthase uses this proton gradient to generate energy in the form of ATP. Complex IV converts oxygen to water (highlighted and boxed in red); thus, measurement of oxygen consumption is a powerful measure of mitochondrial function-mediated oxygen consumption. The amount of oxygen used to make ATP is measured by inhibiting ATP synthase with oligomycin. The maximal amount of oxygen that mitochondria can consume when function increases is measured by “uncoupling” with FCCP; FCCP shuttles protons back to the matrix without any production of ATP, thus uncoupling oxygen consumption from ATP production. Finally, the contribution of non-mitochondrial processes to oxygen consumption is measured by addition of rotenone and antimycin A, which inhibit all electron flow and therefore all mitochondrial oxygen consumption. Any oxygen consumption that occurs in the presence of oligomycin that is mitochondrial can be attributed to proton leak.

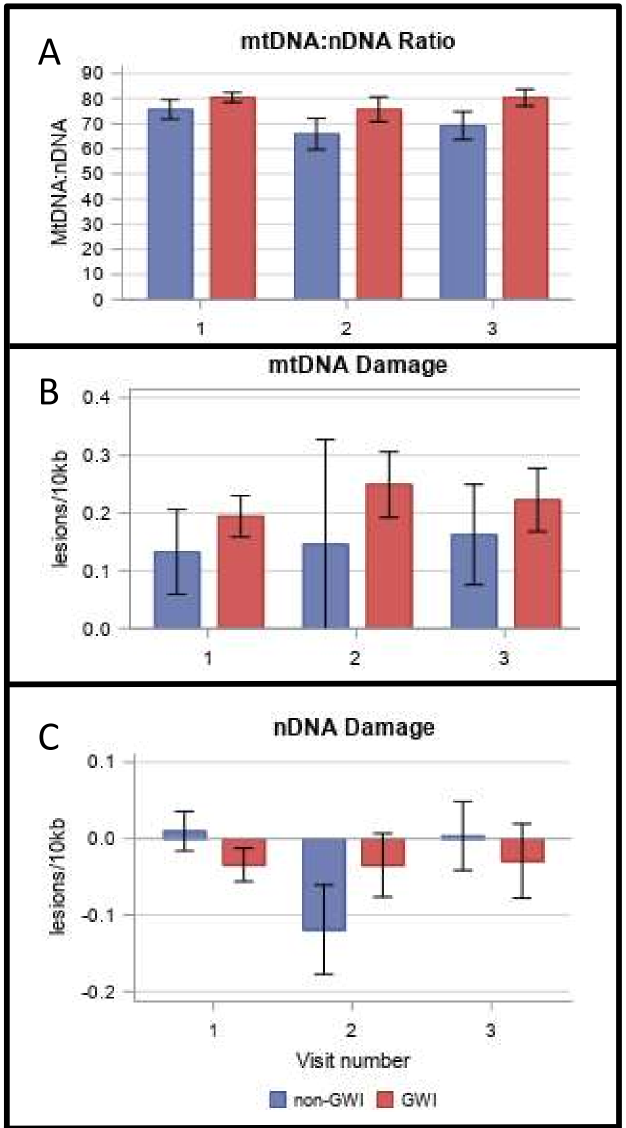

Figure 2. mtDNA copy number is increased in GWI, while mtDNA damage and nDNA damage are unchanged. A) mitochondrial and nuclear DNA CN from whole blood were measured with real-time PCR. B) mtDNA and C) nuclear damage were measured using a long-range quantitative PCR assay.

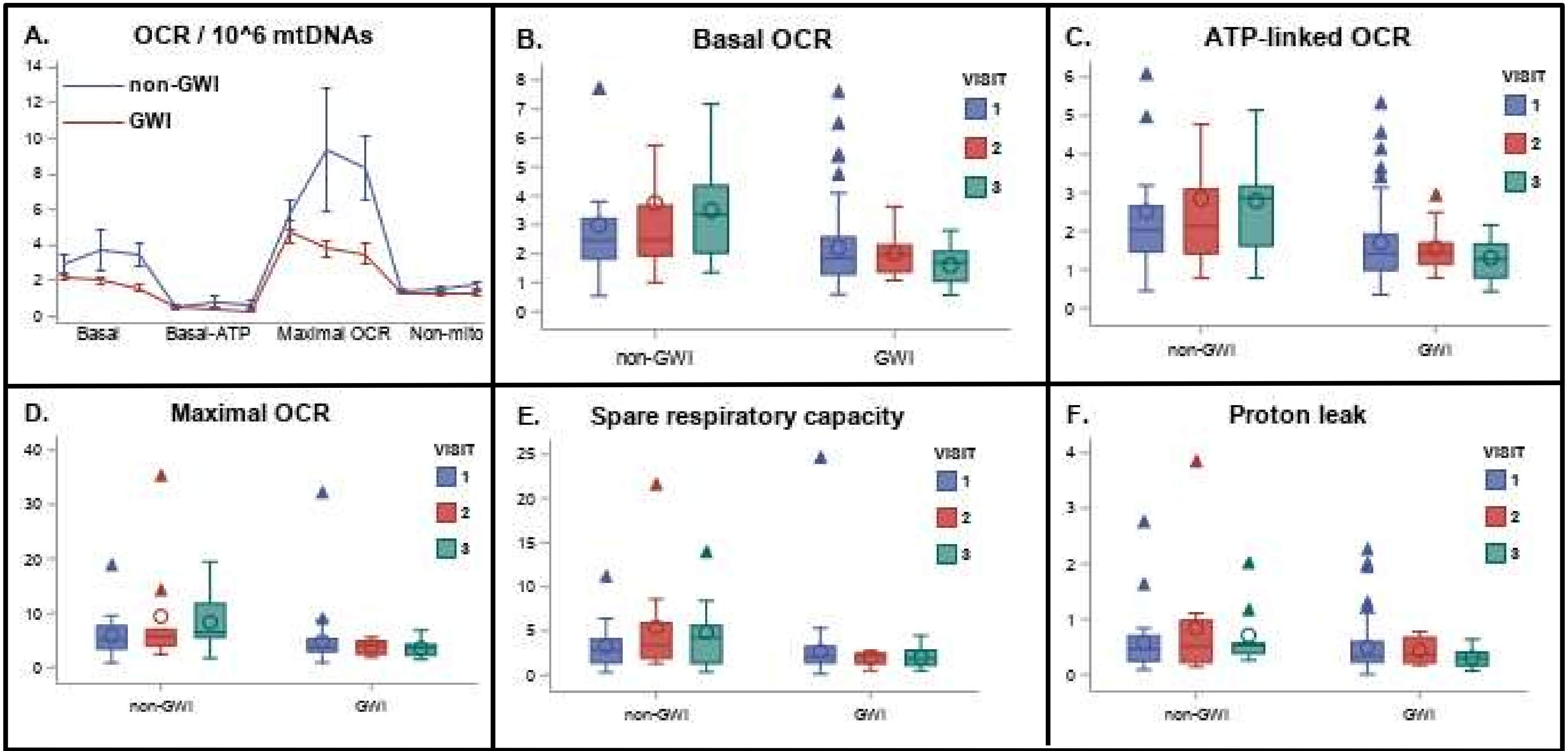

Figure 3. Decreased mitochondrial respiratory function in GWI. A) Average (with standard errors of the mean) oxygen consumption in all samples measured from veterans with and without GWI. Values are normalized to 1,000,000 mtDNA copies. The "basal" reading reflects the amount of oxygen being consumed by cells without the addition of any drugs. ATP-linked OCR is the amount of oxygen consumed upon injection of oligomycin, which inhibits ATP synthesis and therefore blocks oxygen consumption associated with converting ADP to ATP. Maximal respiration is respiration after injection of FCCP, which uncouples mitochondria. Non-mitochondrial respiration is the amount of respiration remaining after the electron transport chain is entirely inhibited with a combination of rotenone and antimycin A. See **Figure 1** for a schematic illustration of the effect of the drugs. Spare respiratory capacity is the differences between maximal and basal; proton leak is the difference between ATP-linked and non-mitochondrial. Specific OCR-related parameters of particular interest are graphed by Kansas status and visit in panels B-F.

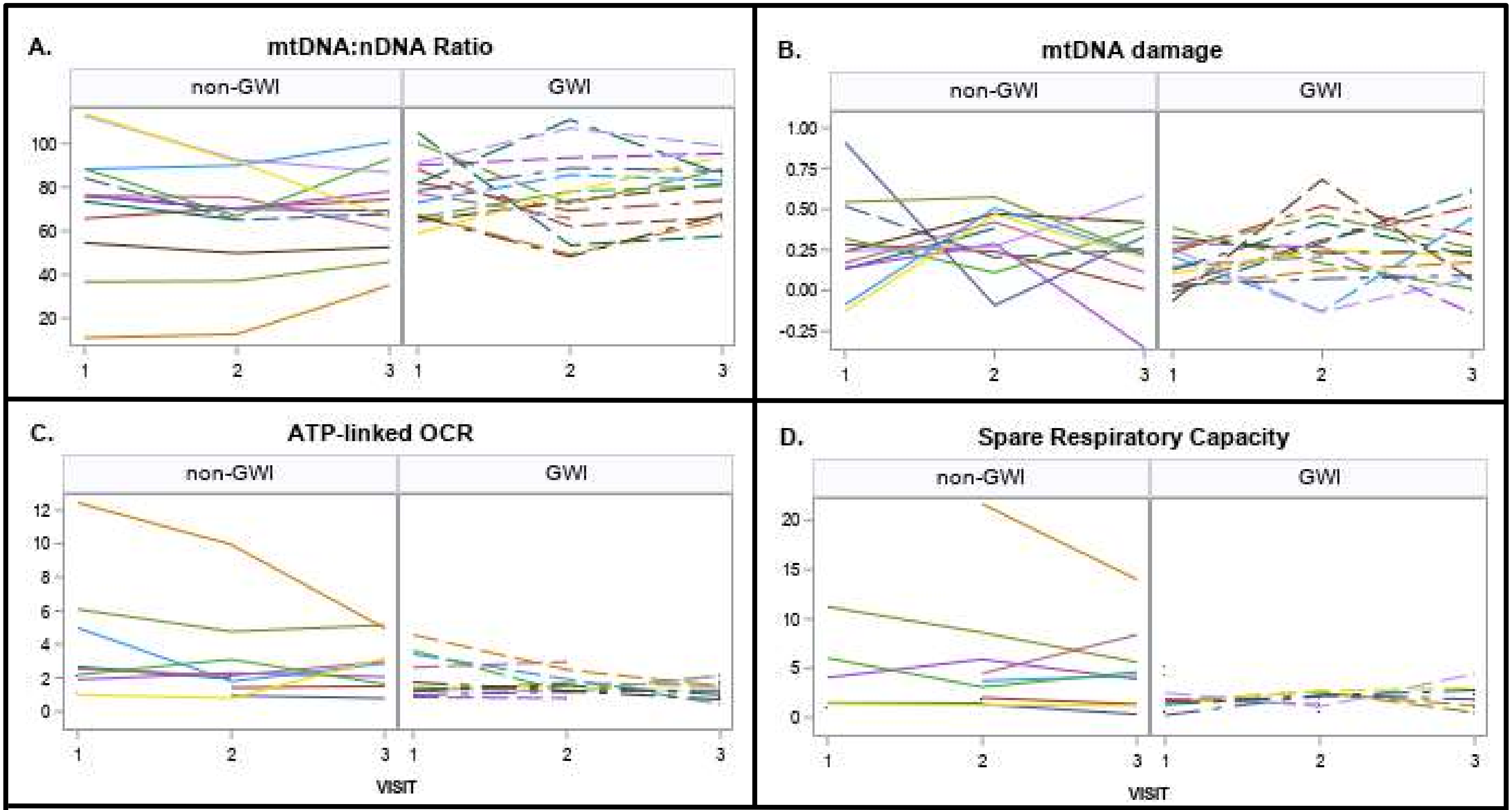

Figure 4. Repeatability of key mitochondrial parameters over time. Note that there is one outlier for mtDNA damage that is not shown, to improve graphical readability; this subject is non-GWI with values of -2.02, -1.94 and -0.44 for visits 1, 2 and 3 respectively.

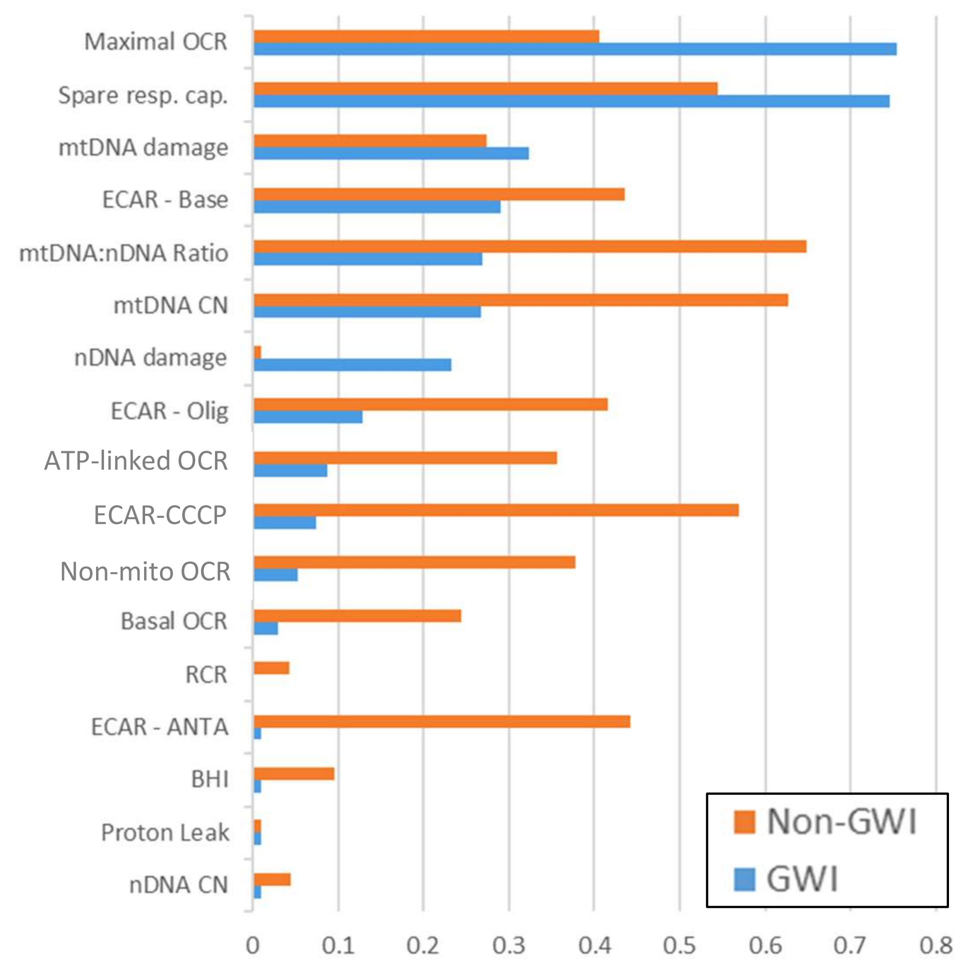

Figure 5. Intraclass Correlation Coefficients by GWI status. For graphing purposes, ICC estimates below zero were set to 0.01.

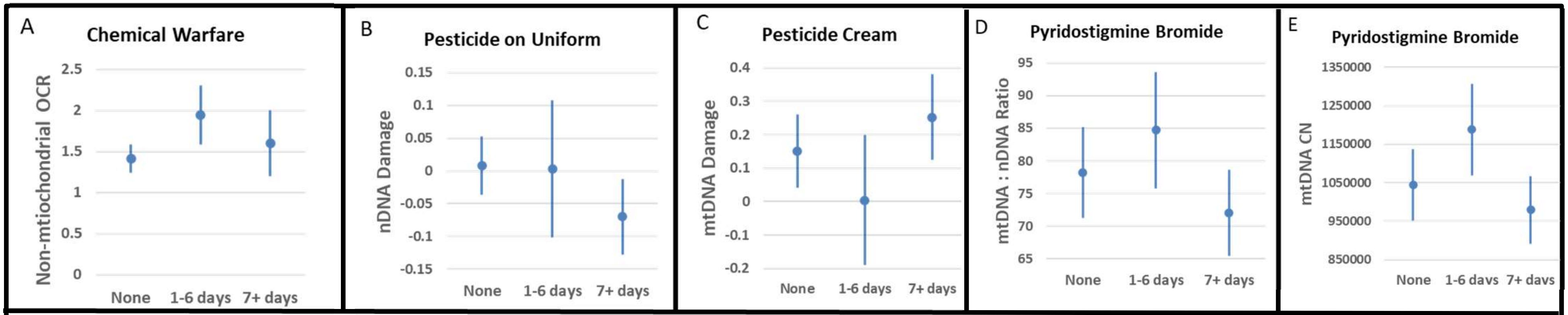

Supplementary Figure 1. Exposures that were associated with mitochondrial parameters after controlling for GWI status. Mean effects and 95% confidence intervals for days of chemical exposures, adjusting for GWI status.

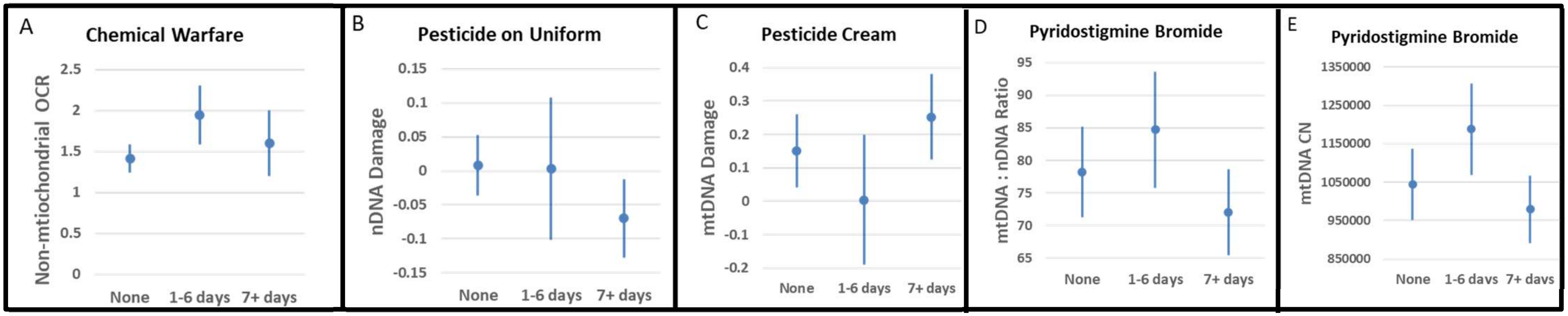
A
Chemical Warfare
Non-mtiochondrial OCR
2.5
2
1.5
1
0.5
0
None
1-6 days
7+ days
B
Pesticide on Uniform
nDNA Damage
0.15
0.1
0.05
0
-0.05
-0.1
-0.15
None
1-6 days
7+ days
C
Pesticide Cream
mtDNA Damage
0.4
0.3
0.2
0.1
0
-0.1
-0.2
None
1-6 days
7+ days
D
Pyridostigmine Bromide
mtDNA : nDNA Ratio
95
90
85
80
75
70
65
None
1-6 days
7+ days
E
Pyridostigmine Bromide
mtDNA CN
1350000
1250000
1150000
1050000
950000
850000
None
1-6 days
7+ days
