# Supplementary material for: Bioenergetic function is decreased in peripheral blood mononuclear cells of veterans with Gulf War Illness
Source: PLoS One. 2023 Nov 1;18(11):e0287412. doi: 10.1371/journal.pone.0287412 (PMC10619881; doi:10.1371/journal.pone.0287412)
Supplement: S1 Checklist — (DOCX) [file pone.0287412.s003.docx]

STROBE Statement—checklist of items that should be included in reports of observational studies

|  | Item No. | Recommendation | Page  No. | Relevant text from manuscript |
| --- | --- | --- | --- | --- |
| **Title and abstract** | 1 | (*a*) Indicate the study’s design with a commonly used term in the title or the abstract | 2 | “In this case-control observational study,…” |
|  |  | (*b*) Provide in the abstract an informative and balanced summary of what was done and what was found | 2 | In this study, we tested multiple measures of mitochondrial function and integrity in a cohort of 114 GW veterans, 80 with and 34 without GWI as assessed by the Kansas definition.  We observed a 9% increase in mtDNA content in blood in veterans with GWI, but did not detect differences in DNA damage. Basal and ATP-linked oxygen consumption were respectively 42% and 47% higher in veterans without GWI, after adjustment for mtDNA amount. We did not find evidence for a compensatory increase in anaerobic energy generation: extracellular acidification was also lower in GWI (12% lower at baseline). |
| Introduction | | | |  |
| Background/rationale | 2 | Explain the scientific background and rationale for the investigation being reported | 4-6 | See Figure 1 |
| Objectives | 3 | State specific objectives, including any prespecified hypotheses | 6 | We tested the primary hypotheses that mtDNA CN would be decreased, mtDNA damage increased, mitochondrial respiration decreased, and ECAR increased, in GWI. We also tested whether recalled exposure to pesticides, chemical or biological agents, and PB would be associated with GWI. Finally, we carried out statistical analyses testing whether the relationship between these mitochondrial parameters and GWI would be affected by gender, exercise, smoking, alcohol use, and body-mass index, parameters shown in previous work to affect mitochondrial function. |
| Methods | | | |  |
| Study design | 4 | Present key elements of study design early in the paper | 6 | “We tested the primary hypotheses that mtDNA CN would be decreased, mtDNA damage increased, mitochondrial respiration decreased, and ECAR increased, in GWI. We also tested whether recalled exposure to pesticides, chemical or biological agents, and PB would be associated with GWI. Finally, we carried out statistical analyses testing whether the relationship between these mitochondrial parameters and GWI would be affected by gender, exercise, smoking, alcohol use, and body-mass index, parameters shown in previous work to affect mitochondrial function.” |
| Setting | 5 | Describe the setting, locations, and relevant dates, including periods of recruitment, exposure, follow-up, and data collection | 5 | “Gulf War Veterans (GV) who deployed in support of Operations Desert Storm and Shield (1990-1991) were recruited between 4/2017 - 4/2019 to participate in this study through our national specialty clinic (New Jersey War Related Illness and Injury Study Center) and the surrounding region using traditional methods (e.g., research flyers, website advertisement, word of mouth) as well as through rosters provided by the Department of Defense’s Manpower Data Center.” |
| Participants | 6 | (*a*) *Cohort study*—Give the eligibility criteria, and the sources and methods of selection of participants. Describe methods of follow-up  *Case-control study*—Give the eligibility criteria, and the sources and methods of case ascertainment and control selection. Give the rationale for the choice of cases and controls  *Cross-sectional study*—Give the eligibility criteria, and the sources and methods of selection of participants | 5 | “GWI case status was determined in accordance with the Kansas GWI Case Definition [47]. In brief, GVs with GWI must endorse moderately severe symptoms in at least three symptom domains (pain, fatigue neurocognitive, skin, gastrointestinal, and/or respiratory), with symptoms first endorsed during or following their Gulf War deployment. GVs who did not meet the GWI case definition served as controls.” |
|  |  | (*b*) *Cohort study*—For matched studies, give matching criteria and number of exposed and unexposed  *Case-control study*—For matched studies, give matching criteria and the number of controls per case |  |  |
| Variables | 7 | Clearly define all outcomes, exposures, predictors, potential confounders, and effect modifiers. Give diagnostic criteria, if applicable | 5-11 | Articulated clearly in these areas |
| Data sources/ measurement | 8* | For each variable of interest, give sources of data and details of methods of assessment (measurement). Describe comparability of assessment methods if there is more than one group |  |  |
| Bias | 9 | Describe any efforts to address potential sources of bias |  | We addressed selection bias by advertising broadly to the veteran population without restrictive exclusion criteria. We addressed recall bias related to exposures by not informing participants that exposures might be associated with mitochondrial measurements. |
| Study size | 10 | Explain how the study size was arrived at |  | The originally proposed sample size of 152 participants (76 per group) was arrived at using power calculations based on preliminary data for mtDNA damage and copy number, assuming an alpha-corrected Type I error rate of 0.016. The preliminary data was ultimately published as part of Chen et al., 2017 https://pubmed.ncbi.nlm.nih.gov/28910366/. |

Continued on next page

| Quantitative variables | 11 | Explain how quantitative variables were handled in the analyses. If applicable, describe which groupings were chosen and why |  |  |
| --- | --- | --- | --- | --- |
| Statistical methods | 12 | (*a*) Describe all statistical methods, including those used to control for confounding | 10-11 |  |
|  |  | (*b*) Describe any methods used to examine subgroups and interactions | 10-11 |  |
|  |  | (*c*) Explain how missing data were addressed |  | We assume missing data were missing at random. Therefore, participants missing any particular variable in a specific analysis were excluded from that analysis |
|  |  | (*d*) *Cohort study*—If applicable, explain how loss to follow-up was addressed  *Case-control study*—If applicable, explain how matching of cases and controls was addressed  *Cross-sectional study*—If applicable, describe analytical methods taking account of sampling strategy | 10-11 |  |
|  |  | (*e*) Describe any sensitivity analyses |  |  |
| Results | | | | |
| Participants | 13* | (a) Report numbers of individuals at each stage of study—eg numbers potentially eligible, examined for eligibility, confirmed eligible, included in the study, completing follow-up, and analysed | 11 | We enrolled 121 veterans, and excluded seven due to potentially confounding health issues. Of the remaining 114, 80 met Kansas criteria for GWI, and 34 did not. 27 GVs returned for a second visit, and 26 for a third visit, allowing us to assess the consistency of measured parameters over time |
|  |  | (b) Give reasons for non-participation at each stage | 11 | We enrolled 121 veterans, and excluded seven due to potentially confounding health issues. Of the remaining 114, 80 met Kansas criteria for GWI, and 34 did not. 27 GVs returned for a second visit, and 26 for a third visit, allowing us to assess the consistency of measured parameters over time |
|  |  | (c) Consider use of a flow diagram | n/a |  |
| Descriptive data | 14* | (a) Give characteristics of study participants (eg demographic, clinical, social) and information on exposures and potential confounders | 12 | Table 1 |
|  |  | (b) Indicate number of participants with missing data for each variable of interest | 12 | Missing data varies depending on the analysis. Please refer to Table 1. In total, 121 veterans were enrolled, and 7 were excluded for confounding health issues. Of the remaining 114 veterans, all reported age, BMI, gender, smoking history, and alcohol history. The number of veterans with missing data varied by covariate: 24 pyridostigmine bromide; 22 pesticide cream; 42 pesticide on uniform; 6 iPAQ; 51 exposure to chemical warfare. For laboratory measurements (i.e., mtDNA, OCR, ECAR), there are 112 veterans with at least one mtDNA measurement (i.e., 2 missing), 98 with at least one ATP (16 missing), and 83 with ECAR measures (31 missing) |
|  |  | (c) *Cohort study*—Summarise follow-up time (eg, average and total amount) |  |  |
| Outcome data | 15* | *Cohort study*—Report numbers of outcome events or summary measures over time |  |  |
|  |  | *Case-control study—*Report numbers in each exposure category, or summary measures of exposure |  |  |
|  |  | *Cross-sectional study—*Report numbers of outcome events or summary measures |  |  |
| Main results | 16 | (*a*) Give unadjusted estimates and, if applicable, confounder-adjusted estimates and their precision (eg, 95% confidence interval). Make clear which confounders were adjusted for and why they were included | 11, 18 | Table 2 and 3 |
|  |  | (*b*) Report category boundaries when continuous variables were categorized |  | Age, BMI and iPAQ scores were categorized. Five age categories were used: 44-49, 50—54, 55-59, 60-64 and 65+. BMI was classified using recommended categories by CDC (normal BMI<25, overweight 25≤BMI<30, obese BMI≥30). iPAQ scores were also categorized per iPAQ protocols into quartiles. |
|  |  | (*c*) If relevant, consider translating estimates of relative risk into absolute risk for a meaningful time period |  |  |

Continued on next page

| Other analyses | 17 | Report other analyses done—eg analyses of subgroups and interactions, and sensitivity analyses | n/a |  |
| --- | --- | --- | --- | --- |
| Discussion | | | | |
| Key results | 18 | Summarise key results with reference to study objectives | 19 | Our results indicate significantly decreased bioenergetic function in PBMCs from veterans with GWI. In particular, we measured decreased oxygen consumption associated with ATP generation as well as decreased extracellular acidification; both are consistent with less availability of energy. This was the case despite a small increase in mtDNA CN in GWI. Thus, our results are consistent with the hypothesis that cellular energetic insufficiency contributes to GWI. We also obtained strong support for a role for chemical exposure in GWI, as previously reported. Finally, we found that GWI was more common among younger veterans. Below, we discuss each of these and other points; we also discuss possible relevance of these results for diagnosis and treatment, limitations, and future directions for research. |
| Limitations | 19 | Discuss limitations of the study, taking into account sources of potential bias or imprecision. Discuss both direction and magnitude of any potential bias | 25 | “Limitations and future directions. As mentioned above, a limitation associated with our results is that it…” |
| Interpretation | 20 | Give a cautious overall interpretation of results considering objectives, limitations, multiplicity of analyses, results from similar studies, and other relevant evidence | 26 | Our results demonstrate significantly decreased bioenergetic function in GWI. Strengths of the study include comprehensive analysis of bioenergetic function and incorporation of normalization of mtDNA copy number in a relatively large cohort of GVs. |
| Generalisability | 21 | Discuss the generalisability (external validity) of the study results | 24 | The urgency of understanding mitochondrial dysfunction in GWI is heightened by the age of these veterans. |
| Other information | |  | | |
| Funding | 22 | Give the source of funding and the role of the funders for the present study and, if applicable, for the original study on which the present article is based |  | This work was supported by the Office of the Assistant Secretary of Defense for Health Affairs through the Gulf War Illness Research Program under Award No. W81XWH-16-1-0663, and supported in part by the Merit Review Award # I01 CX001329 from the United States Department of Veterans Affairs Clinical Sciences Research and Development Service. |

*Give information separately for cases and controls in case-control studies and, if applicable, for exposed and unexposed groups in cohort and cross-sectional studies.

**Note:** An Explanation and Elaboration article discusses each checklist item and gives methodological background and published examples of transparent reporting. The STROBE checklist is best used in conjunction with this article (freely available on the Web sites of PLoS Medicine at http://www.plosmedicine.org/, Annals of Internal Medicine at http://www.annals.org/, and Epidemiology at http://www.epidem.com/). Information on the STROBE Initiative is available at www.strobe-statement.org.
